# Randomised Evaluation of Sleep in Cognitive Impairment Trial (REST)—Protocol for a Feasibility Study of a Digital Cognitive Behavioural Therapy for Insomnia Intervention

**DOI:** 10.3390/ijerph23060695

**Published:** 2026-05-25

**Authors:** Patrick Crowley, Alasdair L. Henry, Mary O’Donovan, Ruth Sheehan, Evelyn Flanagan, Rónán O’Caoimh

**Affiliations:** 1 Department of Geriatric Medicine, Mercy University Hospital, Grenville Place, T12WE28 Cork City, Ireland; 103707938@umail.ucc.ie; 2 Regional Specialist Memory Clinic, Mercy University Hospital, Lancaster Gate Building, Western Road, T12Y63C Cork City, Ireland; maodonovan@muh.ie (M.O.); rusheehan@muh.ie (R.S.); 3 Health Research Board Clinical Research Facility, University College Cork, Mercy University Hospital, T12WE28 Cork City, Ireland; e.flanagan@ucc.ie; 4 Big Health Ltd., London WC1H 9LT, UK; alasdair.henry@bighealth.com

**Keywords:** Alzheimer’s disease, dementia, sleep, insomnia, cognitive behavioural therapy, feasibility, protocol

## Abstract

**Highlights:**

**Public health relevance—How does this work relate to a public health issue?**

- Insomnia is highly prevalent in mild cognitive impairment and dementia and is associated with accelerated cognitive decline, increased morbidity, and healthcare utilisation.
- This study addresses sleep disturbance as a potentially modifiable behavioural risk factor for dementia, a public health priority, using a scalable digital intervention.

**Public health significance—Why is this work of significance to public health?**

- By evaluating the feasibility of a digital cognitive behavioural therapy for insomnia (dCBT-I) in cognitively impaired adults, this trial explores a low-cost, non-pharmacological strategy to improve sleep, while potentially slowing functional and cognitive deterioration.
- The intervention may reduce the reliance on sedative–hypnotic medications, thereby supporting deprescribing initiatives and reducing the fall risk, polypharmacy, and medication-related harm in older adults.

**Public health implications—What are the key implications or messages for practitioners, policy makers, and/or researchers in public health?**

- If feasible, digitally delivered dCBT-I could be integrated into routine memory clinic pathways as a scalable and accessible adjunct to standard dementia care.
- Findings will inform the design of a definitive trial and contribute to emerging dementia prevention and brain health strategies that incorporate sleep as a core intervention target.

**Abstract:**

Dementia is a leading and growing cause of disability worldwide. Insomnia is highly prevalent in mild cognitive impairment (MCI) and dementia and is associated with impaired cognition, functional decline, and reduced quality of life (QoL). Although sleep disturbance represents a potentially modifiable risk factor within the trajectory of cognitive impairment, there have not been many studies conducted to examine the feasibility or preliminary efficacy of digital cognitive behavioural therapy for insomnia (dCBT-I) in this population. The aim of this pilot randomised controlled feasibility study is to evaluate the acceptability, adherence, and potential clinical effects of a multi-component dCBT-I programme (Sleepio) in adults with MCI or mild dementia and comorbid insomnia. Thirty community-dwelling adults aged ≥50 years with established MCI or mild dementia (Mini-Mental State Examination ≥18; Clinical Dementia Rating 0.5–1.0) and insomnia (Sleep Condition Indicator ≤16) will be randomised (1:1) to Sleepio or a wait-list control. Feasibility outcomes include recruitment and retention rates, intervention adherence (completion of ≥4/6 sessions), and acceptability measured using validated usability scales. Secondary outcomes include changes in sleep, mood, QoL, cognition, and function over 10 weeks. Adverse events will be monitored to assess safety. Findings will inform the design of a future definitive trial evaluating digital sleep interventions in cognitively impaired populations. Ethical approval has been granted. The trial is registered at ClinicalTrials.gov (NCT07363928).

## 1. Introduction

Sleep, and receiving the recommended amount (an average of 7 to 9 h) of it, is important for overall physical and mental health for persons at all ages of life but particularly as we age [1,2]. From middle age onwards, a typically U-shaped pattern is seen, with a shorter and longer sleep duration associated with greater cognitive impairment [1]. The prevalence of sleep disturbances including insomnia increases in older age, with increased frailty and greater multi-morbidity [2,3,4]. Insomnia impacts negatively on physical and mental health [5] and is associated with a decline in cognitive performance [6]. Two recent meta-analyses suggest that insomnia increases the risk of developing dementia [7,8]. In addition to epidemiological associations, emerging mechanistic evidence further supports a bidirectional relationship between sleep disturbance and neurodegeneration [9]. Experimental models suggest that sleep plays a critical role in the clearance of neurotoxic metabolites, including β-amyloid and tau proteins, via the glymphatic system [10]. The disruption of slow-wave sleep in particular has been associated with increased amyloid deposition, impaired synaptic plasticity, and neuroinflammation [11]. Human neuroimaging studies similarly demonstrate associations between reduced sleep efficiency, increased sleep fragmentation, and greater amyloid burden [12]. These findings raise the possibility that chronic insomnia may not only be a consequence of neurodegenerative processes but may also contribute to their progression. Further, sleep disturbance is also associated with impaired attention, executive function, and memory consolidation [13], domains commonly affected in mild cognitive impairment (MCI), a prodromal state before dementia, and mild dementia. Therefore, insomnia may exacerbate the existing cognitive vulnerability in this population, compounding functional decline and caregiver burden. Targeting insomnia may thus represent a modifiable risk factor within the trajectory of cognitive impairment.

Medications used to manage insomnia are commonly used but associated with multiple different adverse outcomes including a heightened likelihood of delirium, depression, falls, and even higher mortality [14]. In addition, some research suggests that sleep medications including benzodiazepines and so-called “z drugs” may have detrimental effects on cognitive functioning in this population [15]. Further, a recent meta-analysis suggests that these medications are associated with incident dementia including Alzheimer’s disease [16]. Accordingly, there has been a growing emphasis on avoiding hypnotics among older patients and those with cognitive impairment by identifying and evaluating alternative non-pharmacological interventions for insomnia at all ages but especially in older individuals with cognitive decline including dementia [17]. The role of new pharmacological therapies, including orexin receptor antagonists with potentially improved safety profiles compared to traditional hypnotics, have the potential for use in this population but are yet to be studied in detail [18].

Cognitive behavioural therapy for insomnia (CBT-I) is widely endorsed as a first-line therapy for insomnia for which there is a robust and growing evidence base for an effect on sleep. Despite this, we have not found any studies that have shown an effect on cognition and the majority have been conducted in persons with normal cognition [19,20,21,22]. A large systematic review and meta-analysis of sleep interventions in individuals with cognitive impairment incorporated just two small studies of CBT-I, both of which reported significant improvements in sleep-related outcomes [23]. One study, involving 28 individuals with MCI and comorbid insomnia living in independent accommodation, demonstrated significant benefits across objective as well as subjective sleep measures, as assessed using wrist actigraphy and a screening tool called the Insomnia Severity Index (ISI), respectively [24]. By contrast, the results of the second study, which included 35 participants with MCI recruited from outpatient services, did not reveal improvements in objective sleep parameters, although subjective sleep quality showed significant gains [25]. Importantly, participants in this latter study did not exhibit marked baseline sleep disturbances that could account for the absence of an overall effect in the meta-analysis. In light of the positive findings from the study involving participants with baseline insomnia, further investigation into the use of CBT-I for those experiencing cognitive decline is warranted.

Despite its established efficacy, access to CBT-I remains constrained, due to the costs, limited access, time restraints, and insufficient availability of trained clinicians [26]. Digital delivery formats may help address these limitations by offering advantages such as scalability, lower costs, consistent implementation, and remote accessibility. For older adults who experience mobility difficulties or reside in geographically remote areas, these approaches may reduce barriers to treatment access. Digital CBT-I (dCBT-I) therefore represents a potential alternative to conventional therapist-led CBT-I. Evidence from meta-analyses supports the effectiveness of dCBT-I among adults (without cognitive impairment) [27,28], and more recent studies indicate that it is both feasible and beneficial for older adults [29]. A small mixed-methods investigation involving 12 participants provided the initial evidence for the acceptability of dCBT-I, using the Sleep Healthy Using the Internet for Older Adults Suffering with Insomnia and Sleeplessness (SHUTi OASIS) measure (see NCT03213132), with older individuals diagnosed with MCI [30]. The trial using SHUTi OASIS had several limitations; no patients with dementia were included and it was a single-arm design with a highly selected sample of motivated individuals, with an increased risk of bias [30]. In addition, a non-blinded open-label randomised controlled trail (RCT) assessing feasibility reported that a dCBT-I (Sleepio^TM^ version 2.0) reduced ISI scores among individuals with MCI [31]. Sleepio is a dCBT-I programme that has demonstrated effectiveness in treating insomnia [32,33], leading to improvements in self-reported cognition [34], and has demonstrated efficacy at improving sleep specifically among older patients [35]. Based on its evidence, Sleepio is endorsed by the United Kingdom’s National Institute for Health and Care Excellence or NICE as a first-line intervention for the treatment of insomnia [36]. However, again, none of the patients included had dementia and, as the study was performed entirely remotely, it is likely that participants had more mild deficits and were sufficiently familiar with digital technologies to engage with the intervention in a meaningful way, raising the risk of selection bias.

In addition, although these two studies showed feasibility, questions about digital exclusion, usability challenges, selection bias favouring more motivated patients, and uncertainty over sustained engagement among individuals with cognitive impairment, remain unanswered. Further, caregiver involvement may be necessary for some participants, raising important considerations regarding co-design, accessibility, and support structures. The rigorous evaluation of feasibility parameters, including adherence, usability metrics, and qualitative acceptability, will therefore be critical in determining whether dCBT-I can be successfully implemented in routine clinical practice for this population.

### Aim and Objectives

As previous studies were limited exclusively to individuals with MCI, there is a need to include and study feasibility with patients with mild dementia. While, at these stages, individuals typically retain sufficient insight, learning capacity, and functional independence to engage meaningfully with structured behavioural programmes, it is also important to understand the role of and need for caregivers or supporters in engaging with dCBT-I using a hybrid support model. It is similarly necessary to examine whether such an intervention is acceptable and safe in this population, and to estimate the preliminary effect sizes to inform the design and power calculation of a future definitive (Phase III) trial. Thus, the overall aim of this project is to conduct a pilot randomised controlled feasibility study of a dCBT-I intervention (i.e., Sleepio), in patients attending a university hospital memory clinic with MCI or mild dementia, with the following objectives: (1) to determine participant (patient and study partner if deemed suitable) recruitment, retention, and adherence rates; (2) to assess the usability and acceptability of the intervention by analysing overall participant satisfaction and the frequency of adverse events; (3) to examine pre/post-intervention changes in sleep; (4) to examine the effects on psychosocial health including mood; and (5) to examine pre- and post-intervention changes in cognition and function.

## 2. Materials and Methods

### 2.1. Design

REST is an investigator-blinded, randomised, controlled, two-arm, parallel feasibility study. Participants will be allocated in a 1:1 ratio to either the intervention group or a true wait-list control group through a centrally managed, computer-generated randomisation process. An asymptotic maximal randomisation strategy, with a maximum tolerated imbalance of three, will be employed. The study coordinator will not be blinded to group allocation, as this is necessary to deliver training on the intervention to participants in the intervention arm. In contrast, data collectors and controllers, statisticians, and site investigators will remain blinded to treatment assignment. Participants will be randomised in a 1:1 ratio using a computer-generated sequence. Allocation concealment will be ensured using sequentially numbered, opaque, sealed envelopes.

Baseline variables recorded will include demographic information (such as age, sex, ethnicity, employment status, and education status), lifestyle factors (such as smoking history, level of physical exercise, caffeine, and alcohol consumption), medical co-morbidities, and regular medications. The principal investigator, an attending/consultant physician, will adjudicate on medical inclusion and exclusion criteria. Assessment of outcome measures will take place at baseline and post-intervention, which will be set at 10 weeks post randomisation. Participants assigned and randomised to the intervention group will continue to receive treatment as usual in addition to the intervention described in the next section. Those assigned to the wait-list control group will be told that they will receive the intervention at the end of the 10-week study period. They will receive ‘treatment-as-usual’ during the study period.

### 2.2. Intervention and Comparator

Sleepio is a personalised and fully automated dCBT-I intervention programme designed specifically to manage and treat insomnia based on established evidenced cognitive behavioural techniques. It incorporates cognitive and behavioural interventions in conjunction with sleep hygiene education and relaxation techniques. The intervention is delivered via an automated digital platform that uses underlying algorithms to personalise the content, guidance, and support provided to each participant. This tailoring is informed by individual responses to a baseline sleep assessment questionnaire, as well as ongoing data captured through daily sleep diary entries completed throughout the programme. In this way, the intervention dynamically adapts to the user’s sleep patterns and reported difficulties, allowing for a more individualised and responsive therapeutic experience.

The programme comprises six structured sessions, each typically lasting between five and twenty minutes. Following completion of the initial session, subsequent sessions are released sequentially at weekly intervals, thereby encouraging gradual engagement with the intervention over time. While the programme is designed to be completed over a minimum period of six weeks, user engagement patterns suggest that most participants take approximately 9–10 weeks to complete all sessions. Importantly, there is no strict time limit imposed on completion, and, once sessions are unlocked, they remain accessible for repeated viewing, enabling participants to revisit content as needed to reinforce learning and support behavioural change. Participants and/or their study partners will be offered daily SMS messages/emails to support compliance and adherence.

Because the intervention is delivered digitally, participants’ level of digital literacy will be evaluated prior to randomisation using the Mobile Device Proficiency Questionnaire [37]. All individuals assigned to the intervention group will then receive a training/orientation session to support their use of the Sleepio programme. They will also be provided with clear written instructions on how to access and use the programme. Participants with poor digital literacy will receive an initial one-hour-long one-to-one training session with one of the study investigators. A basic briefing will be offered to participants who are deemed digitally literate. If participants wish to include a study partner to support their participation in the study, or if it is clear to the investigator that they will require such support, the study partner where needed will attend and participate in the training/orientation session.

### 2.3. Participants and Setting

Patients will be community dwelling adults with insomnia and established MCI or mild dementia attending a large university-hospital-associated outpatient memory clinic affiliated with the Mercy University Hospital in Cork City in the south of Ireland called the Regional Specialist Memory Clinic (RSMC), Cork, Ireland.

#### 2.3.1. Inclusion Criteria

Participants will be deemed eligible for inclusion provided they are aged 50 years or older and meet criteria for insomnia, defined as a score of ≤16 on the Sleep Condition Indicator (SCI) [38] (an 8-item measure scored from 0 to 32 points, with scores ≤ 16 suggesting insomnia), aligned with DSM-5 diagnostic criteria. Eligible participants must have a prior clinical diagnosis of MCI or mild dementia made by an attending physician, with dementia staged as mild (based on the Reisberg Functional Assessment Staging Tool stages 3 or 4 [39]) and MCI defined according to Petersen’s criteria [40]. Cognitive status will be further confirmed by the Mini-Mental State Examination (MMSE), a widely-used test of cognition which is scored from 0 to 30 points, with higher scores indicating better cognition [41] and the Clinical Dementia Rating (CDR), which is a commonly-used clinician rated measure of global symptom severity and burden in persons with cognitive impairment [42]. To participate, those with MCI/mild dementia must score ≥18 on the MMSE and ≥0.5 to 1.0 on the CDR. Participants must have access to the internet via a computer, tablet, or smartphone for the duration of the intervention, and possess sufficient physical and sensory capacity (including vision and hearing ability) to engage with the intervention, as determined by the study team. Digital literacy will be assessed and supported separately, see above. Additional inclusion criteria include being English-speaking as the Sleepio digital CBT-I program is only currently available in English, living in the community (i.e., not in a nursing home or other institution), and having the capacity to provide informed written consent as judged by the principal investigator.

#### 2.3.2. Exclusion Criteria

Participants will be excluded if they have a diagnosed sleep disorder other than insomnia, as defined by the International Classification of Diseases. Individuals who have received CBT-I within the previous six months will also be excluded. To ensure inclusion of participants with mild stage impairment (MCI or dementia), those with an MMSE score <18/30 at enrolment will be ineligible. Exclusion criteria further include severe depression (e.g., requiring hospital admission within the preceding 12 months or recent psychiatric outpatient review), as well as unstable depression, anxiety, or panic disorders, defined by changes in psychotropic medication within the preceding three months. Individuals with other major neuropsychiatric conditions (including psychosis, schizophrenia, bipolar disorder or other mania disorder, and epilepsy, or other seizure disorders), or those with ongoing substance or alcohol misuse, will also be excluded. Additional exclusions include significant physical, sensory, or medical conditions that may impede participation, as judged by the principal investigator, as well as planned surgery or hospitalisation during the study period. Participants deemed medically unfit to continue by the investigator will not be enrolled. Finally, individuals who have had recent (within three months) changes in medications that may affect sleep or cognition, including hypnotics, anxiolytics, antidepressants, antipsychotics, cognitive enhancers (e.g., cholinesterase inhibitors or memantine), stimulants, decongestants, opioid analgesics, β-blockers, certain pulmonary medications, or melatonin, will be excluded.

### 2.4. Recruitment

Consecutive patients attending the RSMC Cork will be screened for eligibility using continuous recruitment during the study window. Patients who meet the predefined inclusion criteria will be invited to participate in this feasibility study, in line with recommendations for pilot trials. Eligibility screening will be undertaken by the principal investigator or a delegated member of the research team, all of whom are experienced clinicians working within the participating memory clinics. The target of the study is to recruit a total of 30 patients over a six-month recruitment period. A formal sample size calculation has not been undertaken, as the primary objective is to assess feasibility outcomes; however, this sample size is considered adequate to evaluate key parameters such as recruitment, retention, and intervention adherence, and to inform the design and sample size estimation of a future definitive RCT. If available and deemed by the study investigators to be required, study partners, defined as family members or close friends, will also be recruited to provide support to participants and to facilitate engagement with the intervention throughout the study period.

### 2.5. Consent Process

Fully informed and written consent will be required from each participant before enrolling them in the study. Capacity to provide informed consent will be assessed and confirmed by an attending consultant physician, ensuring that all participants have the ability to understand the nature, purpose, and potential risks and benefits of the research. Only individuals deemed capable of providing valid consent will be included. Where applicable, additional written consent will also be sought from study partners, defined as caregivers, family members, or close friends, who are willing to participate in a supportive role. These individuals will be informed about their role within the study and their involvement will be contingent on their voluntary agreement to participate. All study procedures will be conducted in accordance with applicable data protection legislation, including the European Union’s General Data Protection Regulation (a.k.a. GDPR), with appropriate measures in place to ensure the confidentiality, secure storage, and controlled access of all personal data.

### 2.6. Outcome Measures

These feasibility thresholds will be predefined a priori based on published recommendations for pilot and feasibility studies together with adherence data from comparable digital behavioural interventions.

#### 2.6.1. Primary Outcomes

1. Recruitment of the target number of 30 participants during a six-month study period.
2. Retention of ≥70% of participants throughout the study period. Retention of ≥70% was selected as a pragmatic benchmark consistent with previous complex behavioural and digital health interventions. Retention can be particularly challenging in online interventions including digital sleep treatments, where a large systematic review reported a mean study discontinuation rate of 23% across e-health interventions [43]. Similarly, digital CBT-I studies have reported variable retention rates, including rates of approximately 66% in some online insomnia interventions [44], while retention rates approaching 80% have been reported in trials of Sleepio involving participants without cognitive impairment [33]. Given the additional challenges associated with retention in persons with cognitive impairment, a retention threshold of ≥70% was considered indicative of acceptable feasibility.
3. Adherence rate of ≥66%, defined as completion of four out of the six sessions included in the intervention. Completion of four or more sessions has previously been used to define treatment completion in digital CBT-I studies, including trials of Sleepio where adherence approaches 80% in those with normal cognition [32]. However, as mean adherence rates in digital mental health interventions are approximately 60% [45], and because cognitive impairment may present additional barriers to sustained engagement, an adherence threshold of ≥66% was considered appropriate.
4. Acceptability of the intervention, which will be assessed using both the System Usability Scale (SUS) [46] and Usability Metric for User Experience Lite (UMUX-Lite) [47]. The SUS is a well-established, validated, brief, ten-item Likert-scale measure designed to provide an overall assessment of perceived usability that is scored on a range from 0 to 100, with scores greater than 68/100 considered to be of average usability [46]. In this study, our target is that ≥70% of the sample will score ≥ 70/100 on the SUS as an indicator that it is usable and acceptable. The UMUX-Lite is a shorter, Likert-type instrument with two items also scored from 0 to 100 that likewise evaluates user satisfaction with a system or product to infer usefulness and usability. While there is no precise cut-off point, scores ≥ 70/100 are considered acceptable, albeit similar to the SUS; there is no strict cut-off. Hence, in this study, we will also target ≥70% scoring ≥70/100 as a marker of acceptability.
5. Participant engagement will be assessed using objective platform metrics including the number of logins, completion of sleep diary entries, session completion rates, and overall duration of programme use. Feasibility thresholds were predefined based on published recommendations for pilot and feasibility trials and comparable behavioural intervention studies.

#### 2.6.2. Secondary Outcomes

All secondary outcomes are exploratory and will not be powered. These will be obtained in order of hierarchy according to sleep, psychological, cognitive, and functional domains:

1. Changes in sleep, which will be assessed by the following: changes in SCI scores [38] and Insomnia Severity Index (ISI) scores [48], a validated 7-item instrument to assess self-reported insomnia severity over the previous two weeks. Scores range from 0 to 28, taking a cut-off of ≥8 as the sub-threshold for insomnia and scores ≥ 15 indicating clinical insomnia [48].
2. Changes in psychosocial health, which will be assessed pre- and post-intervention by examining the following:
  - Patient Health Questionnaire 8 (PHQ-8) [49], a valid diagnostic and severity measure for depressive disorders;
  - Generalised Anxiety Disorder 7 (GAD-7) [50], a widely-used and validated measure for assessing generalised anxiety disorder;
  - Euroqol EQ 5D Visual Analogue Scale [51], which records subjective assessment of health-related quality of life on a 100-point vertical visual analogue scale on which the endpoints are labelled ‘the best health you can imagine’ and ‘the worst health you can imagine’;
  - Dementia Quality of Life Instrument (DEMQOL) [52], a validated 29-item measure for assessing self-reported quality of life in people with dementia.
3. Changes in cognition and function, which will be assessed pre- and post-intervention by the MMSE [39], CDR Scale (global score) [42], and Alzheimer’s Disease Assessment Scale—Cognitive Subscale (ADAS-Cog) [53], a validated assessment of cognition involving 11 tasks and scores ranging from 0–70, Disability Assessment for Dementia [54] (a 40-item assessment of functional ability involving personal and instrumental activities of daily living in people with dementia), and the British Columbia Cognitive Complaints Inventory (BC-CCI) [55] (a brief 6-item assessment of self-reported cognitive difficulties involving concentration, memory, articulation, thinking, and problem-solving over the previous seven days, with scores ranging from 0 to 18, with higher scores indicating greater perceived cognitive impairment).

### 2.7. Statistical Analysis

In accordance with Consolidated Standards of Reporting Trials (CONSORT) Guidelines [56], descriptive statistics will be used to report participants’ baseline characteristics and details of participant flow, including recruitment, retention, and adherence to the intervention. Categorical variables will be described using frequencies and percentages. Regarding continuous variables, histograms and boxplots will be used to evaluate the distribution of the data and to identify any outliers or potential errors. The mean and standard deviation will be used to describe the typical value and spread in the sample but, where data is not normally distributed, the median and interquartile range will be used. All tests of significance will be two-sided and conducted at an α = 0.05 level for statistical significance.

Feasibility outcomes (e.g., recruitment rates, retention, adherence to the intervention, and data completeness) will be summarised descriptively. These outcomes will be used to inform the design and sample size estimation of a future definitive trial. Regarding the bespoke questionnaire for acceptability and usability of the intervention, frequency distributions of the categorical data in both study groups will be compared using chi-square tests or Fisher’s exact tests, as appropriate. For continuous secondary outcomes measured at baseline and post-intervention, between-group differences at follow-up will be analysed using analysis of covariance (ANCOVA) within a linear regression framework to compare changes between study groups from baseline to post-intervention with respect of the secondary outcomes. Adjustments will be made for covariates associated with the intervention and the outcome as appropriate (for example, age, sex, baseline levels of sleep disturbance, and cognitive impairment). Model assumptions will be assessed using graphical methods, including inspection of residual plots to evaluate linearity, homoscedasticity, and normality of residuals. Multicollinearity will be assessed using variance inflation factors (VIFs).

**Figure 1:**
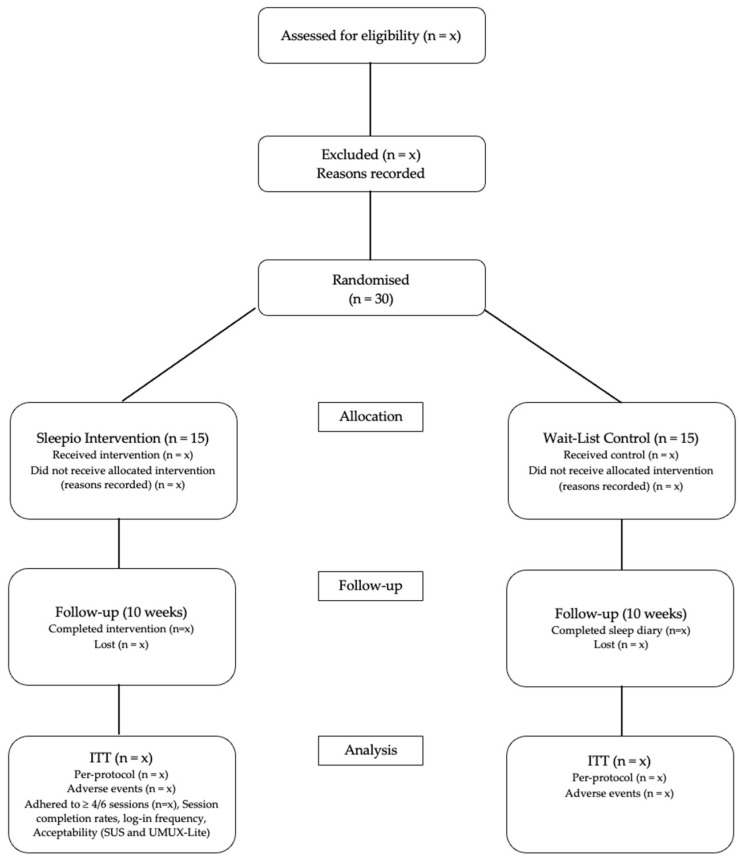
CONSORT flow diagram for the REST pilot randomised controlled feasibility study. Participants are randomised (1:1) to digital cognitive behavioural therapy for insomnia (Sleepio) or wait-list control and followed for 10 weeks. Analyses will be conducted using intention-to-treat (ITT) and per-protocol approaches. SUS = System Usability Scale (SUS), UMUX-Lite = Usability Metric for User Experience Lite.

Analyses will be conducted according to the intention-to-treat (ITT) principle, including all participants with available outcome data in the groups to which they were randomised. Missing outcome data will be assumed to be missing at random, and no imputation will be performed for the primary analysis in this feasibility study. Mixed-effects models using all available observed data will be employed under this assumption that data are missing at random. The extent and patterns of missing data will be reported descriptively. No formal adjustment for multiple comparisons will be applied, as secondary analyses are exploratory and hypothesis-generating. Consistent with methodological recommendations for pilot studies, a sample of 30 participants is considered sufficient to estimate key feasibility parameters and generate variance estimates for a future definitive trial. Feasibility outcomes will be presented with 95% confidence intervals. Exploratory between-group effect sizes (Cohen’s d) will also be calculated to inform future sample size calculations. The overall study flow is illustrated in Figure 1.

### 2.8. Safety Monitoring

Adverse events including transient daytime somnolence, mood deterioration, or worsening sleep will be monitored throughout the study. Events will be documented, graded according to severity, and assessed for relatedness to the intervention. Participants may be withdrawn and the study stopped if continued participation is deemed unsafe.

### 2.9. Patient and Public Involvement (PPI) Statement

This protocol is informed by interviews conducted with people living with cognitive impairment and their carers regarding the acceptability and usability of the Sleepio intervention. The protocol was reviewed by the PPI panel of Dementia Trials Ireland.

### 2.10. Ethics and Registration

Ethical approval for this feasibility study has been granted by the Clinical Research Ethics Committee of the Cork Teaching Hospitals on the 17 December 2024, CREC references: ECM 4 (i) 22 October 2024; ECM 5 (4) 12 November 2024; and ECM 3 (ww) 10 December 2024. The study has been registered on clinicaltrials.gov, registration number: NCT07363928 (updated 23 January 2026). The study will be conducted in accordance with the Declaration of Helsinki.

### 2.11. Dissemination

The results of this research will be disseminated through publication in peer-reviewed journals and presentation at scientific conferences and patient organisations. Findings will be shared with clinicians in memory services, general practitioners, and allied health professionals to support integration of sleep interventions into routine care. Lay summaries will be provided to participants, caregivers, and advocacy groups, and disseminated via institutional and digital platforms. Given the scalability of digital cognitive behavioural therapy for insomnia, findings will be relevant to health service planners and policy makers, including within Irish and European dementia strategies. Results will inform the design of a future definitive RCT.

## 3. Discussion

This pilot randomised controlled feasibility study will analyse the feasibility, acceptability, and preliminary efficacy of a dCBT-I intervention to improve sleep, psychosocial health, and cognitive function in participants with MCI and mild dementia. The results will inform the design of a future definitive RCT of the intervention in this population, if it is shown to be feasible. If feasible and ultimately effective, benefits associated with the intervention possibly include direct short-term effects on sleep, thereby increasing subjective energy, reducing fatigue, and improving quality of life. In the medium term, given the evidence for CBT-I including Sleepio as a proven non-pharmacological intervention for sleep [32,33,34,35], the reliance on pharmacological therapies with their associated side-effects [57,58,59] could be reduced. The long-term benefits are more speculative and may include the potential to slow cognitive and functional decline in this population, which would, in turn, reduce the rates of morbidity, mortality [60], and institutionalisation [61]. However, given that this is a feasibility study, any hypotheses testing will need to be conducted as part of an adequately powered definitive trial.

From a public health perspective, insomnia represents a highly prevalent and modifiable risk factor within ageing populations, albeit one that is often considered neglected [62]. Dementia is now one of the leading causes of disability and dependency worldwide, with profound societal and economic consequences. Even modest delays in cognitive decline at a population level could translate into substantial reductions in adverse health outcomes [61]. Sleep disturbance contributes not only to cognitive dysfunction but also to increased risk of falls [63], frailty [64], and mood disorders [65], each of which independently increases healthcare utilisation among older adults. Addressing insomnia may therefore generate multidimensional benefits extending beyond cognition alone. In particular, improvements in sleep continuity and quality may reduce daytime somnolence and improve attention, executive function, and mood, thereby enhancing functional independence and participation in daily activities. From a systems perspective, interventions that preserve autonomy and delay institutionalisation are of critical importance in the context of rapidly ageing populations.

Furthermore, the pharmacological management of insomnia remains common despite the well-documented risks in older adults, including falls, delirium, and polypharmacy-related complications. A safe and effective non-pharmacological alternative could contribute to deprescribing initiatives and reduce medication-related adverse events. This aligns with international strategies aimed at improving medication safety and reducing potentially inappropriate prescribing in older people. Another benefit is that, as it is a low-cost intervention (£45 per person was recommended by National Institute for Health and Care Excellence [NICE] as cost-effective in improving sleep/treating insomnia), it is affordable for patients and healthcare systems. CBT-I, while evidenced and beneficial, is not without potential harms, including transient daytime sleepiness, which is largely due to the sleep restriction therapy component of CBT-I. Participants will be warned of this potential side-effect prior to enrolling in the study.

### Limitations

A limitation includes the single (investigator)-blinded design. Participants in the wait-list control group could hence alter their behaviour, potentially influencing study results and introducing bias. In addition, given the multiple assessments, there is a risk of the test burden on participants resulting in fatigue, inaccurate results including type 1 error inflation, or, indeed, withdrawal from the study [66]. This said, understanding this is a component of feasibility testing. To overcome this, the outcomes have been ordered in a hierarchy emphasising the feasibility measures over the secondary outcomes, and, if patients do experience fatigue, the assessments can be reduced to minimise this by adhering to the set order. Nevertheless, the potential for multiplicity and type I error inflation will be acknowledged and findings interpreted precisely and cautiously [67]. These exploratory analyses are intended principally to inform outcome selection, sample size estimation, and the design of a future definitive trial, and no formal hypothesis testing is intended. Another potential limitation is that, although some structured support is considered necessary in this feasibility study design, the scalability of this level of support in routine practice will require further evaluation. The multiple assessments also risk missing data. In this study, the missing outcome data will be assumed to be missing at random (MAR), and no formal imputation procedures will be undertaken. Instead, the extent and patterns of missing data will be described descriptively. This is appropriate, as many of the ways to impute missing data such as ‘last observation carried forward’ increase the risk of bias [68,69]. Finally, while the target sample size is modest and no formal sample size calculation was performed, there is no accepted guideline for feasibility studies; most select between 15–20 per arm with a widely used ‘rule of thumb’ of 12 [70]. This is in keeping with most feasibility studies, where the median sample size was found to be n = 30 [71].

The results of this feasibility study, and any subsequent definitive RCT, will add to the evidence for the use of CBT-I as, to date, there have not been many studies conducted examining this intervention in persons with cognitive impairment and, to our knowledge, none in dementia [72]. A recent systematic review and meta-analysis found that, despite the high levels of acceptability, adherence was not systematically measured in studies of CBT-I, an important gap in the context of digital interventions [72]. As CBT-I delivery expands, identifying the barriers and facilitators to engagement and sustained use will be key to translating the efficacy into a real-world benefit [73]. They may also have important public health implications. A simple, effective, and generalisable intervention for insomnia that can potentially slow cognitive decline will impact positively on healthcare systems as both are common problems at the population level. Low-cost interventions like Sleepio are particularly important in a global health context, where countries have limited health budgets. The potential for an online intervention to provide remote access to target more vulnerable/isolated patients was highlighted during the COVID-19 pandemic [74]. This study will also provide evidence for incorporating sleep modification into primary prevention studies of complex interventions designed to slow or prevent dementia, such as the original *FINGER* intervention study (of diet, exercise, cognitive training, vascular risk monitoring, and social stimulation in those with subjective cognitive decline) [75] and the ongoing *World-Wide Fingers* study multi-country network [76], which currently does not include a sleep component. Clinically, the findings will be generalisable to clinical settings nationally and internationally. They will, for example, be immediately applicable to brain health clinics as the European Academy of Neurology Brain Health Strategy has recognised sleep as a determinant of brain health [77].

## 4. Conclusions

In conclusion, the results of this feasibility study will have important implications for the design and conduct of a future Phase III randomised controlled trial to determine whether dCBT-I can improve sleep and attenuate cognitive and functional decline in people with MCI or mild dementia. By evaluating key feasibility parameters, including recruitment, retention, adherence, and acceptability, this study will provide essential data to inform the trial methodology, outcome selection, and sample size estimation for a definitive study. Beyond its methodological contribution, this research addresses insomnia as a common and modifiable risk factor within cognitively impaired populations. If shown to be feasible and ultimately effective, a scalable, low-cost digital intervention such as dCBT-I could be integrated into routine memory clinic pathways and broader models of dementia care. This has important implications for reducing the reliance on pharmacological treatments, improving quality of life, and potentially delaying the progression to more advanced stages of the disease. At a population level, identifying accessible interventions that target both sleep and cognitive health may contribute meaningfully to public health strategies with the goal of reducing the burden of dementia in ageing societies.

## Data Availability

Data will be made available upon requests made to the corresponding author.
